# Epidemiological Pattern of Injuries in Iran; a Nationwide Review of Seven Million Emergency Department Admissions

**Published:** 2017-01-08

**Authors:** Mohammad Haji Aghajani, Mashyaneh Haddadi, Soheil Saadat

**Affiliations:** 1 Department of Cardiology, Shahid Beheshti University of Medical Sciences, Tehran, Iran.; 2 Injury Prevention and Safety Promotion Department, Ministry of Health and Medical Education, Tehran, Iran.; 3 Sina Trauma and Surgery Research Center, Tehran University of Medical Sciences, Tehran, Iran.; 4 e-Health Department, Virtual School, Tehran University of Medical Sciences, Tehran, Iran.

**Keywords:** Epidemiology, wounds and injuries, epidemiological monitoring, multiple trauma, mortality, accident prevention

## Abstract

**Introduction:**

Globally, it is estimated that around 5.8 million people die annually as result of injuries, which causes 10% of all deaths and 16% of disability adjusted life years lost worldwide. This study aimed to determine the epidemiology of injuries in emergency departments in Iran.

**Method:**

This cross sectional study was carried out using national injury surveillance data registry from 21 March 2009 to 20 March 2014.

**Results:**

7,176,344 patients with the mean age of 27.5 ± 17.8 years were registered to 657 EDs (70.6% male). Road Traffic Crash (RTC) was the most common cause of injury (31.0%) followed by hit (28.2%) and fall (10.1%). While roads were the commonest place of injuries, 34.0% of patients have been injured at home. More than 90% of injuries were unintentional. Assault and suicide attempt were causes of injury in 5.6% and 3.9% of patients, respectively.

**Conclusion:**

This paper addresses where prevention measures are most urgently needed and offers insights which could be useful for injury prevention programs in Iran and other developing countries.

## Introduction

Globally, it is estimated that around 5.8 million people die annually as result of injuries, which causes 10% of all deaths and 16% of the disability adjusted life years (DALY) lost worldwide (1). Injury-related disease burden is expected to rise over the next 20 years and road traffic injuries, suicide and falls which are known as 9^th,^ 15^th^ and 21^st^ leading causes of death are respectively estimated to rise to 5^th^, 12^th^ and 16^th^ cause of death by 2030 (2). Ninety percent of all injury-related deaths occur in low and middle-income countries, whose limited data collection is hampering planning and implementation of preventive measures (3). Injuries are an important challenge for the health system and account for 28% of the DALY lost (21,572) in Iran (4). Iran is in the 5^th^ place for road traffic mortality rate globally, and has the highest mortality rate in Eastern Mediterranean region (5). Although death is the most notable measure of injury, it represents just the tip of the iceberg and to address the problem, data of both fatal and nonfatal injuries are required as non-fatal injuries are quite common and impose huge medical expenses on the population (6). Injury surveillance is the ongoing systematic collection, analysis, interpretation and dissemination of data for planning, implementing, monitoring and evaluation of preventive interventions (7). The absence of reliable estimations on the scale and pattern of injury morbidity and mortality is a major barrier to successfully implement preventive strategies in many low and middle-income countries (8, 9). This study aims to determine the epidemiological pattern of injuries during five years using the national injury surveillance data registry.

## Methods

***Study design and setting***

This cross sectional study was designed to evaluate the epidemiological pattern of injuries among almost all trauma patients presented to the emergency departments (ED) of university hospitals in Iran, between 21 March 2009 and 20 March 2014, using an ED based injury surveillance data registry. The study was approved by ethical committee of Ministry of Health and researchers adhered to all ethical principles of Helsinki Declaration and patient data confidentiality during the study period.

***Data gathering***

To monitor the frequency of fatal and non-fatal injuries on an ongoing basis, identify high-risk groups requiring specific interventions; monitor and evaluate interventions for effectiveness, and produce reports to assist in planning and resource allocation, an ED based injury surveillance data registry system has been established in the Iranian Ministry of Health and Medical Education in 2004. The system has been further developed in 2005 by including all medical university hospitals (10). 

The data gathering process commences when injured patients arrive at an ED. Information is registered on the medical records by the medical staff and no medical doctors are involved. At the end of stay in the ED, data are added to a paper based questionnaire and then into the electronic offline software which is installed in most public university hospitals. 

For hospitals in which the software is not installed, the paper based data is converted into electronic format and retained by the corresponding medical university. Quality control is performed by injury prevention experts from universities and the ministry of health (11).

University hospitals are obliged to register all admitted injured patients; however, we cannot ignore possibility of underreporting in some cases. Therefore, we did not use the data to estimate injury incidence rate in the Iranian population. 

The dataset includes demographic data (age, sex), injury characteristics (time, place, mechanism), and patients’ outcome at the end of ED stay. The injury characteristics were registered according to WHO Injury Surveillance Guideline.

Data of the present study were extracted from the afore-mentioned national registry, using census sampling.

***Statistical analysis***

Statistical analysis was performed using SPSS version 18. Continuous variables are presented as mean ± standard deviation (SD) and discrete ones as frequency and percentage. 

## Results

**Figure 1:**
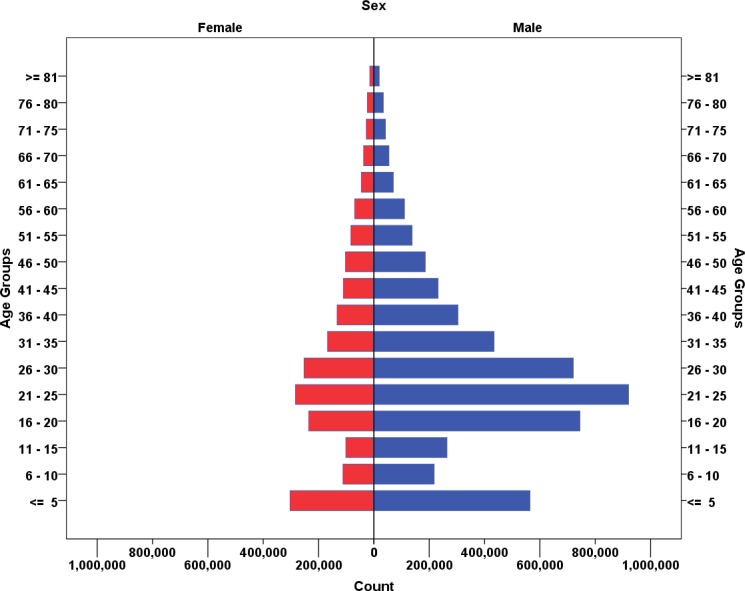
Age and sex distribution of the injured patients.

7,176,344 patients had been registered to 657 EDs all around the country. Mean age of the injured patients was 27.5 ± 17.8 years (0, 102) and 70.6% of the cases were male. Figure 1 presents age and sex distribution of the injured patients.

***Injury characteristics***

**Figure 2:**
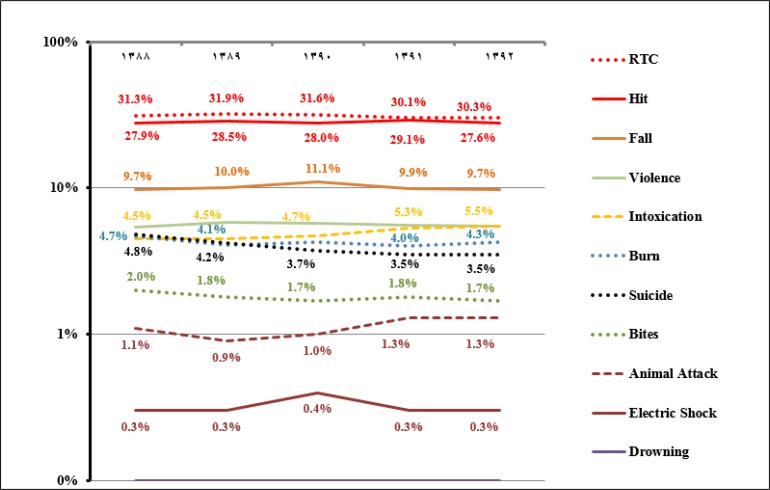
The relative frequency of causes of injury during the study period (Dotted lines represent decreasing trend, dashed lines represent increasing trend and solid lines are used for steady trend), y axis is in logarithmic scale; RTC: road traffic crash

Table 1 summarizes the frequency of injury causes among the studied patients. Road traffic crash (RTC) was the most common cause of injury (31.0%) followed by hit (28.2%) and fall (10.1%). Among the patients injured due to RTC, 962,045 (43.3%) were car occupants, 862,429 (38.8%) motorcyclists, and 399,253 (18.0%) were pedestrians. Frequency of violence and suicide attempt were 402203 (5.6%) and 277563 (3.9%), respectively. The relative frequency of intoxication and animal attacks as cause of injury showed a raise during the study period; while burn, suicide, RTC and bites presented with a decreasing trend (Figure 2). 

**Figure 3:**
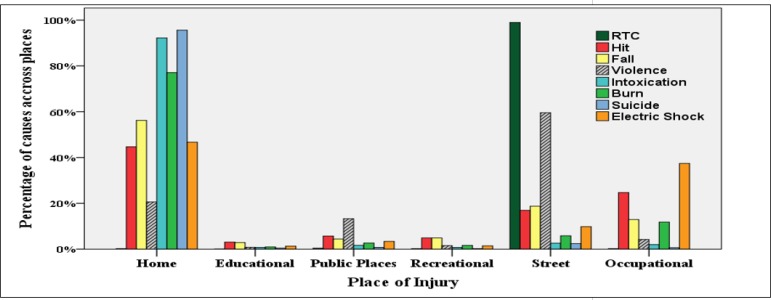
Distribution of common causes of injury according to place of occurrence; RTC: road traffic crash

**Figure 4:**
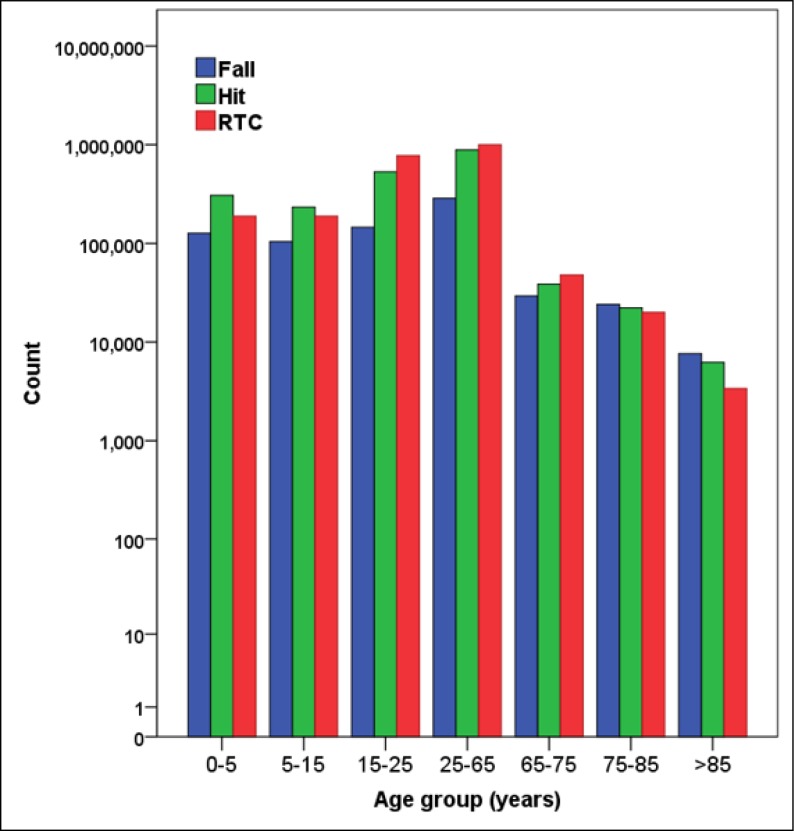
The leading causes of injury in different age groups (y axis is in logarithmic scale).

 2,440,703 (34.0%) of patients had been injured at home and 700,789 (9.8%) at work places. Figures 3 and 4 show the distribution of common causes of injury according to place of occurrence and the age group of the injured patients. Most injuries had happened in urban areas (79.9%) followed by rural (12.8%) and intercity (5.8%). Roadside was the most common place of injury with 3,008,072 (41.9%) cases. Summer (28.8%) was the commonest season for emergency admission of injured patients followed by spring (26.6%), and winter (20.6%).

***Male to female ratio***

**Figure 5:**
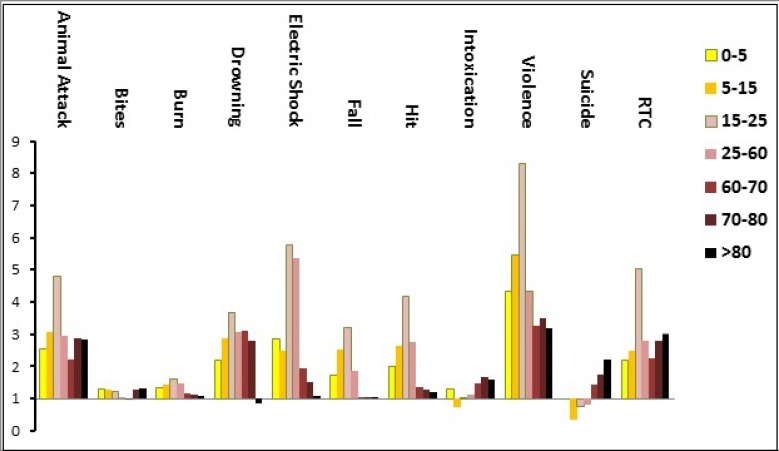
Male to Female ratio of patients in different age groups according to the cause of injury.

Figure 5, displays the male to female (M/F) ratio in different age groups according to the cause of injury. Overall, the highest M/F ratio was seen in patients injured due to violence and in 15-25 years age group (8.3). The ratio was 3.3 for violence related injuries that took place in the street and public places, and 0.7 for violence related injuries that occurred at home, regardless of age groups. 

***Mortality rate***

**Figure 6:**
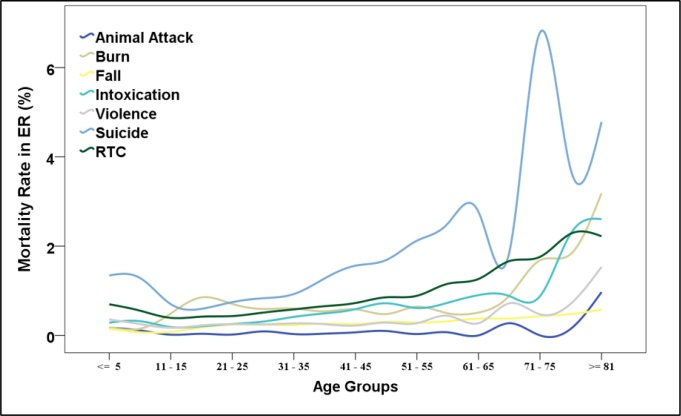
Mortality rate in emergency department per 100 cases according to the age group; RTC: road traffic crash

Mortality rate in ED was 490, 443, 386, 383 and 387 per 100,000 cases in consecutive study years. It was highest in suicide attempts and in the elderly (Figure 6). Mortality rate in ED for injuries that occurred in urban, rural and intercity areas were 295, 702 and 1291 per 100,000 cases, respectively.

## Discussion

Iran is one of the 77 countries that have a national injury surveillance system (ISS). Less than half of high and middle-income countries, and less than a quarter of low-income countries have established a nationwide ISS (5). The current Iranian national ISS yet needs to be improved; however, it can help in understanding the epidemiology of severe injuries that require medical care throughout the country.

Iran with a population of 78.8 million in 2015 and land area of 1.6 million square kilometers (12) experiences the age standardized injury mortality of 74.9 per 100,000 population (13).

According to findings of this study, all age groups have been affected by injuries with the highest frequency belonging to young males. A similar pattern has been reported in previous studies from Iran, Saudi Arabia, and China. (11, 14, 15). However, case fatality of the injured in ED showed a raise in the elderly. This indicates that preventive programs should encompass all age groups; while elderly injured patients need specialized and intensive care in the ED. This should be considered in triage and referral protocols as old injured patients would better be referred to level-one trauma centers.

RTC continues to be an increasing source of morbidity and mortality worldwide with the developing countries worst hit due to rapid unplanned industrialization and urbanization. Our study, further highlights this fact by introducing RTC as the most common cause of injury, like previous studies (11, 16, 17). Iran was experiencing an increasing trend in RTC fatality and injury rates until 2005 (18). The death rate due to RTC hit the global record of 44 per 100000 population in 2005 (19). Conversely, from 2006 the trend was reversed (18-20) and annual RTC fatality rate dropped to 21.6 in 2014 according to official National Road Traffic Safety Status report by Ministry of Road & Urban Development. The relative frequency of RTC compared to other causes of injury showed a decreasing trend in our study. This could be a result of the preventive programs for RTC in line with the international framework of the decade of action on road safety. Iran has been committed to reduce road fatalities by 10% annually, according to the 5^th^ five year development plan beginning in 2011. Health sector aims to control injuries and promote safety through a balanced approach based on primary, secondary and tertiary prevention. Increasing the number of emergency medical service (EMS) stations, initiating the air ambulance services, renovating EMS fleet as well as improving the quality of pre and in-hospital care are among health sector programs that contribute to reducing road fatalities. 

Among road users, car occupants had the highest ED admission rate, followed by motorcyclists and pedestrians. This is similar to reports from comparable studies (11, 21). Our study did not include on-site mortality cases; however, a national study that included all RTC mortalities for a decade, reported that about half of traffic mortality cases have been car occupants followed by motorcyclists and pedestrians (22). Although pedestrians are the most vulnerable group in RTC, speeding makes car occupants the group most commonly referred to ED due to RTC. As seen in Table 2 the proportion of car occupants is highest in RTCs occurred in intercity areas where speeding is possible, and least in urban areas where law enforcement and traffic jam makes it difficult to speed up. Limiting the speed of driving according to the guidelines of vision zero (23) or sustainable safety program (24) should be considered by local authorities to reduce the number of road users injured due to RTC (25).

**Table 1:** Cause of injury distribution

**Cause**	**Number (%)**
**RTC**	2225123 (31.0)
**Hit**	2026468 (28.2)
**Fall**	724506 (10.1)
**Violence**	402203 (5.6)
**Poisoning**	353999 (4.9)
**Burn**	305066 (4.3)
**Suicide attempt**	277563 (3.9)
**Bites**	127316 (1.8)
**Animal Attack**	81871 (1.1)
**Electric Shock**	23707 (0.3)
**Drowning**	4960 (0.1)
**Others**	623153 (8.7)
**Unknown**	409 (0.0)
**Total**	7176344 (100.0)

RTC: road traffic crash.

**Table 2:** The distribution of road traffic crash (RTC) injured patients according to the region and type of road user

**Region**	**Position**		
	**Pedestrian**	**Car occupant**	**Motorcycle rider**
**Urban**	354670 (22.5)	565392 (35.9)	653974 (41.5)
**Rural**	33568 (11.5)	111021 (38.1)	146910 (50.4)
**Intercity**	9537 (2.8)	278151 (81.0)	55535 (16.2)
**Unknown**	1478 (9.9)	7481 (50.0)	6010 (40.1)
**Total**	399253 (18.0)	962045 (43.3)	862429 (38.8)

Data were presented as number and percentage; the position of 1396 RTC injured patients was not known.

Motorcycle riders are the second most common injured group in RTC. A population based study has shown that although 19.7% of households owned a motorcycle in the capital of Iran, the attributable risk of RTIs due to motorcycles was 63.9% in 2008 (26). Speed limit, using safety helmet and traffic law enforcement should be considered to protect both motorcyclists and pedestrians from RTC.

Although RTC was a major cause of injury in all ages, it appeared as the leading cause of injury in 15-75 years age groups. This is in line with other studies and points out the fact that RTC is clustered in active age groups. Similarly, fall is a major cause of injury in all ages but it overrides other causes in ages older than 75. This pattern has been reported in population based studies as well (6). This is partly due to a decrease in RTC rate in the elderly as a result of reduced social activity and partly due to increased risk of falling in this population due to imbalance and osteoarthritis as a result of aging (6). Population based interventions are required to increase the awareness of families about strategies to prevent the elderly from falling and keep old people as physically active as possible to improve their balance and body muscle mass. Moreover, there is a need to monitor the adverse effect of medications they are using and also make suitable environmental modifications to prevent fall or limit its consequences. Prevention and treatment of osteoporosis should be considered in every elderly who refers to health centers. 

Roadside was found to be the leading place of injury occurrence followed by home. The findings of this study are consistent with those of Rastogi et al. (27), which is due to higher incidence of RTI compared to other injuries. Home being the second most common place of injury could be explained by the amount of the time spent at home and the vast variety of activities taking place there. Majority of suicide attempts, intoxication, burns, fall and electric shock injuries occur at home. Therefore, to reduce the incidence of above-mentioned injuries, interventions are needed. Interventions should aim to increase the safety knowledge of families, raise the willingness of parents to pay for safety, and also provide affordable safety devices for low income families. Households that take care of children and elderly should be of priority in safety improvement interventions.

Violence was the cause of injury in 5.6% of ED admissions. In a similar study of ED admission from 2005 to 2008, violence was accountable for 5.2% of injuries (11). There is need for effective violence prevention interventions in the public to reduce this avoidable injury. Streets are the place that majority of violence related injuries take place, followed by home and public places. As a result, violence prevention programs should be aimed at these places. Violence in street and public places generally take place between strangers, usually incidentally and involve mainly males. This type of violence could be prevented by police interference and redesigning public areas to reduce the stress and conflict of society. However, violence prevention at home needs different strategies because this type of violence takes place between familiar people and involves females 2.4 times more than the violence occurred in public places and streets. This type of violence accounts for about 20% of violence injuries and needs special consideration. Health sector has developed a policy paper on domestic violence to address intentional injuries. Since the survivors of domestic violence not only need alternatives to returning home, risk assessment, and referrals for counseling, and legal services but also medical care including assurances that they were not at fault for the battering, a policy was developed in 2012 as a joint program between Ministry of Health and Medical Education and WHO office in Iran to sensitize and increase health care providers’ awareness of domestic violence.

Suicide accounted for 3.9% of ED admissions. Sharif-Alhoseini et al. showed a similar proportion for suicide attempts in their study that included ED admissions from 2005-2008 in Iran (28). There is a strong stigma for suicide in Iran, which may result in underreporting of suicide attempts in ED. However, suicide is an avoidable injury and unlike most other injuries, there are medical approaches for its prevention. Therefore, health sector may be able to control this type of injury by providing efficient mental health care to vulnerable patients. The fact that most suicide attempts took place at home, points out an opportunity for prevention, and highlights the importance of family oriented interventions in facing suicide attempt of a household member. Suicide attempt has been more common in females aged five to sixty years, in contrast to most other injuries. 

Therefore, young and middle aged females under 60 years old are in priority for suicide prevention. Besides preventability, there is another reason to allocate resources to suicide prevention: it has the highest case fatality in ED in most age groups. Health care personnel should be trained to identify people at risk of suicide attempt and health departments should establish programs to support at risk people and their families as well as health system staff to deal with a case at risk of suicide attempt. A training and service manual is prepared and implemented in university hospitals to increase the knowledge of EMS staff, change their attitude, and be used as a guideline to approach aggressive, criminal and suicidal cases.

Work places are another common site of injury that account for about ten percent of ED admissions. There are occupational safety offices in medium and large work sites that supervise adherence to safety regulations in Iran. However, small workshops do not benefit from such a professional safety promotion service and there is need for improvement in these sites.

Although 72.8% of the Iranian population lives in cities in the year 2015, 79.9% of ED admissions due to injury had happened in urban areas. It is not clear whether higher presentation of urban patients is due to higher injury incidence in urban areas or as a result of limited access of rural population to EDs. However, the higher injury fatality in rural area EDs indicates the possibility of under-presenting moderate injuries from rural areas. In other words, patients injured in rural areas refer to ED only in very severe instances compared to urban patients and this may reflect inequality in trauma care. Although, based on the 3rd five year development plan (2000) the government had become responsible for developing trauma system to provide optimum care for trauma patients free of charge, this system has not been implemented yet and only road traffic injured patients’ care is free of charge. Based on the Health Evolution Plan (2014) and changes in out of pocket payments, medical care has become more affordable for traumatic patients in rural areas. Also, because of the long distances between hospitals in rural areas, injured patients who suffer from life threatening situations are referred to the nearest health care center to be stabilized and then referred to designated hospitals based on their medical requirements. In order to address different coverage of EMS in urban and rural areas, remote and outreach areas, health sector scaled up the air ambulance system for better coverage in rural areas.

In terms of seasonality, summer was the commonest season for emergency admission of injured patients followed by spring. This could be partly explained by the increased outdoor activity in the summer that increases the risk of fall, drowning and sport related injuries. On the other hand, RTC was the most common cause of injury and an increase in RTC due to increased road traffic for leisure purposes in spring and summer would reflect in the total number of injuries. Most educational institutes are closed in the summer. Therefore, students who had had to spend almost always in the school would engage in a lot of activities during the summer and they are not necessarily prepared for safety of leisure in this season. There is a need to increase the safety awareness of students before summer vacations and also improve the safety preparedness of the community before summer. The traffic police, road traffic maintenance organization, EMS and Red Crescent take special effort to enforce traffic regulations and increase their road side capacity during the summer. Moreover, health sector runs seaside rescue programs during this season for Caspian Sea. However, there are too many rivers and small pools to be covered by this program. Moreover, there is not any fall prevention program during the summer season, when people may climb trees to harvest. There is also need for animal attack and insect bite prevention programs before beginning of warm seasons. Educational institutes are best places to raise the safety knowledge of students, as a group who will involve in sport and leisure activities more than others. Media can also play a crucial role. Safe community movement is a community based approach that aims to reduce injuries and deaths by bringing together partners to tackle safety issues affecting the community. This approach encourages and supports aspiring communities to deliver local solutions for local concerns through a community-based coalition. At the moment, Iran has 35 designated members in the international safe community network and there is a national commitment to expand the program (29).

As seen in Figure 1, injuries have been more common in males in all age groups, especially in young ages. Meanwhile, the highest M:F ratio was seen in patients injured due to violence and in 15-25 age group. Males may get physical in this age group more than females. This points out the need for anger and bullying behavior control and interpersonal conflict management programs for this age-sex group that could be taught in high schools. These educations are not provided as part of routine training in Iranian schools. High school students may also benefit from educations aimed to raise knowledge and improve the skill against substance abuse that may in turn reduce their involvement in violence, especially in male schools.

The M:F ratio was the least in bites and burn injuries as well as intoxication. While some studies indicate burns were more common in females (30), others indicated slight male dominance for burns (31-34) and poisoning (56%) (35). 

There was a downward trend in case fatality among patients admitted to EDs. The case fatality in 2013 was 21% lower than 2009. This is an evidence of improvement in trauma care in Iran. Obligatory current medical education (CME) programs aimed to improve the quality of trauma care, implementing hospital trauma quality improvement programs such as running morbidity & mortality conferences and fair and equitable distribution of emergency medicine physicians may have played a role in decreasing case fatality of traumatic patients admitted to EDs. Moreover, a significant growth in Emergency Medicine (EM) residency programs boosted the number of EM specialists now staffing EDs throughout the country. These programs should be spread out more to improve the quality of trauma care in outreach areas. The disaster and emergency management center is going to implement an ongoing Preventable Trauma Death (PTD) Study that aims to monitor PTD in all university hospitals and provide prompt feedback to local health authorities, as another quality monitoring and improving program.

This study has some limitations. Injury-related deaths that occurred on the scene of trauma, or following transfer to other wards, an intensive care unit or operating theatre were not included in this study. Moreover, data on external causes of injury, ICD codes, causes of death, injury severity scores, and vital signs were not available since these data are usually recorded in trauma registry systems and not in the ISS. More information is needed on the burden of disabilities due to injuries that can’t be extracted from the current ED based System. 

## Limitations:

We were not able to study the severity of injuries as well as the quality of care. This needs to be addressed in further studies.

## Conclusion:

This paper addresses where prevention measures are most urgently needed and offers insights which could be useful for injury prevention programs in Iran and other developing countries.
